# Modification of Adenosine196 by Mettl3 Methyltransferase in the 5’-External Transcribed Spacer of 47S Pre-rRNA Affects rRNA Maturation

**DOI:** 10.3390/cells9041061

**Published:** 2020-04-24

**Authors:** Olga Sergeeva, Philipp Sergeev, Pavel Melnikov, Tatiana Prikazchikova, Olga Dontsova, Timofei Zatsepin

**Affiliations:** 1 Skolkovo Institute of Science and Technology, Skolkovo, 121205 Moscow, Russia; 2 Serbsky National Medical Research Center for Psychiatry and Narcology, Kropotkinsky Lane 23, 119034 Moscow, Russia; 3 Department of Chemistry, Lomonosov Moscow State University, 119992 Moscow, Russia

**Keywords:** RNA modification, RNA methyltransferase, rRNA processing

## Abstract

Ribosome biogenesis is among the founding processes in the cell. During the first stages of ribosome biogenesis, polycistronic precursor of ribosomal RNA passes complex multistage maturation after transcription. Quality control of preribosomal RNA (pre-rRNA) processing is precisely regulated by non-ribosomal proteins and structural features of pre-rRNA molecules, including modified nucleotides. However, many participants of rRNA maturation are still unknown or poorly characterized. We report that RNA m6A methyltransferase Mettl3 interacts with the 5′ external transcribed spacer (5′ETS) of the 47S rRNA precursor and modifies adenosine 196. We demonstrated that Mettl3 knockdown results in the increase of pre-rRNA processing rates, while intracellular amounts of rRNA processing machinery components (U3, U8, U13, U14, and U17 small nucleolar RNA (snoRNA)and fibrillarin, nucleolin, Xrn2, and rrp9 proteins), rRNA degradation rates, and total amount of mature rRNA in the cell stay unchanged. Increased efficacy of pre-rRNA cleavage at A’ and A0 positions led to the decrease of 47S and 45S pre-rRNAs in the cell and increase of mature rRNA amount in the cytoplasm. The newly identified conserved motif DRACH sequence modified by Mettl3 in the 5′-ETS region is found and conserved only in primates, which may suggest participation of m6A196 in quality control of pre-rRNA processing at initial stages demanded by increased complexity of ribosome biogenesis.

## 1. Introduction

Ribosome biogenesis is vital for the cell. Basic features of ribosome biogenesis are conserved among all eukaryotes, yet ribosomal RNA (rRNA) synthesis and ribosome assembly pathways in human are considerably more complex due to the increased size of ribosomes. Dysregulation of ribosome biogenesis is associated with diseases such as Treacher Collins syndrome (TCS), Shwachman-Bodian-Diamond syndrome (SBDS), dyskeratosis congenita, 5q syndrome, and Diamond-Blackfan anemia (DBA) [1], while augmented ribosome biogenesis contributes to cancer development [2]. The initial stage of ribosome biogenesis (pre-rRNA processing) involves a complex pattern of precise cleavage events, with the participation of several non-ribosomal proteins (nucleases, helicases, etc.) and small nuclear RNAs. Ribosome biogenesis consumes a large portion of energy in the cell and the rate of this process is regulated in a cell cycle-dependent manner. However, many participants of the pre-rRNA cleavage process are still unknown or poorly characterized. Quality control of rRNA processing is precisely regulated and it seems likely that the specificity of the cleavage is largely determined by definite structural features of pre-rRNA molecules, including nucleotide modification. During ribosome biogenesis many nucleotides in functionally important regions of the rRNA precursors are modified either by snoRNA complexes or by enzymes, to improve quality control of maturation.

According to the MODOMICS database of RNA modification pathways, among the 171 nucleotide modifications in RNA, 72 are various methyl modified nucleotides [3,4,5]. The most abundant methylated RNA nucleotide is N6-methyladenosine (m6A). Approximately 0.1–0.4% adenosines in total cellular RNA are N6-methylated, which corresponds to around 50% of all methylated ribonucleotides [6,7]. The m6A is a dynamic modification that can be added and removed, thus allowing fine tuning of RNA stability and binding to protein partners during cell metabolism. A number of proteins are involved in the regulation of this modification: “Writers” (m6A methyltransferases), “erasers” (m6A→A “demethyltransferases”), and “readers” (effectors recognizing m6A) [8]. The most studied m6A “writer” is Mettl3 methyltransferase [9], which mainly targets mRNA [10], while two main “erasers”, demethylases FTO and ALKBH5, control the amount and distribution of Mettl3-mediated modifications [8]. The m6A in the 5′ untranslated region (5′-UTR) of mRNA participates in the control of cap-independent translation [11,12], while m6A in the 3′-UTR determines the lifetime of mRNA [13]. Presence of m6A modification can lead either to mRNA stabilization [14] or promote mRNA degradation [15]. Additionally, m6A is crucial for splicing regulation [16], alternative polyadenylation [17], and mRNA nuclear export [18], and can alter RNA structure and folding [19,20]. Other m6A methyltransferases like CAPAM can also modify mRNAs [21]. Despite all the published information about the roles of m6A modification in mRNA biology, its functions in other eukaryotic RNA are not well studied. Previously, functions of m6A were well characterized in bacterial rRNA, stabilization of secondary rRNA structure (for example, m6A1618 and m6A2030 in 23S rRNA) or antibiotic resistance (m6A2058 in 23S rRNA) [22,23,24]. Recently, two m6A rRNA methyltransferases, Mettl5 and ZCCHC4, were discovered. They are responsible for m6A modification of 18S and 28S rRNA, respectively, which influence on ribosome assembly, translation, and finally cell proliferation [25,26].

Although many essential steps of ribosome biogenesis are well described, initial stages of human 47S pre-RNA processing are not described in detail. Alternative pathways and limited uncovered protein participants of endo- and exonucleolytic cleavages in 5′-ETS, all these issues complicate understanding the functional role of the initial stages in pre-rRNA processing. Here we report a new target of Mettl3 methyltransferase (adenosine 196 in the 5′-ETS of the 47S pre-rRNA) and the study of its role in rRNA maturation. We demonstrated that significant RNAi-mediated knockdown of Mettl3 results in the increase of pre-rRNA processing rates leading to a slight increase of cytoplasmic amounts of 18S, 28S, and 5.8S rRNA as well as monosomes. As quantities of the main known participants of 47S pre-rRNA processing, such as snoRNA U3, U8, U13, U14, and U17, and fibrillarin, nucleolin, RRP9, and XRN2 proteins, were not changed in our study, we do believe that the observed phenotype is driven by the lack of m6A196 in 47S pre-rRNA. Based on sequence similarities, we demonstrated that the identified m6A modification in 47S pre-rRNA is primate-specific and might be an additional feature for quality control of pre-rRNA processing demanded by the increased complexity of ribosome biogenesis.

## 2. Materials and Methods

### 2.1. The Small Interfering RNAs (siRNA) Description and Lipid Nanoparticles (LNP) Formulation

We designed six chemically modified siRNAs (Supplementary Table S1) targeting human Mettl3 sequences (NCBI Genbank accession code NM_019852.5). The siRNA were selected to avoid off-target activity based on several known criteria [27,28,29]. Candidate 19-mer sequences were aligned against RefSeq mRNA database and estimated for their off-target binding capability. In particular, siRNA were ranked based on the number/positions of the mismatches in the seed region, mismatches in the nonseed region, and mismatches in the cleavage site position. The resulting sequences were further filtered to avoid known miRNA motifs and immune stimulatory sequence motifs [28]. Introduction of 2′-OMe pyrimidine nucleotides in siRNA further reduced immune response and off-target effects [28,30] and together with single 3′-internucleotide phosphorothioates increased stability against nucleases. Potency and efficacy of siRNAs were studied by transfection lipofectamine RNAimax (Thermo Scientific, Waltham, MA, USA) in HEK293 cells followed by quantitative PCR (qPCR) analysis after 24 h (Supplementary Figure S1A). The control siRNA targets the Firefly Luciferase gene (control).

The most efficient siRNA and control siRNA were formulated in lipid nanoparticles (LNP) as previously described [31,32]. Briefly, siRNA were dissolved in a 10 mM citrate buffer (pH 3.0) to a final concentration of 0.4 mg/mL. Lipid components (C12-200 [33], 1,2-distearoyl-sn-glycero-3-phosphocholine (DSPC) (Avanti Polar Lipids (Alabaster, AL, USA), 850365), cholesterol (Sigma-Aldrich (St. Louis, MO, USA), C8667) and 1,2-dimyristoyl-sn-glycero-3-phosphoethanolamine-N-[methoxy(polyethylene glycol)-2000] (ammonium salt) (PEG-lipid) (Avanti Polar Lipids, 880150) were dissolved in ethanol and mixed at a 50:10:38.5:1.5 (C12-200:DSPC:cholesterol:PEG-lipid) mol:mol ratio to the final lipid concentration 8.83 mg/mL. The water solution of siRNA and the ethanol solution of lipids (3:1, *v*:*v*) were mixed in the microfluidic NanoAssemblr device (Precision NanoSystems, Vancouver, BC, Canada) at a 10 mL/min total flow rate, according to the manufacturer’s recommendations and as described earlier [32]. The final ratio of siRNA:C12-200 after mixing is 1:5 (*w*:*w*). The resultant LNP suspension was dialyzed against 500 volumes of PBS (Thermo Scientific 10010023) at pH 7.4 in 20,000 MW cut-off dialysis cassettes (Thermo Fisher Scientific, 66012) overnight and filtered through a PES syringe filter (0.2 µm pores) (Corning (Corning, NY, USA), 3915). Particle size analysis was carried out using a Zetasizer Nano ZSP (Malvern Panalytical, Malvern, UK), according to the manufacturer’s protocol. The siRNA entrapment efficiency was determined using the Quant-iT™ RiboGreen^®^ reagent (Thermo Fisher Scientific R11491), as described earlier [32]. 

### 2.2. Cell Culture and Transfection

The experiments were performed in human embryonic kidney cells HEK293 (ATCC cell biology collection^®^ CRL- 3216). Cells were cultured in Dulbecco’s Modified Eagle Medium (DMEM) medium (Thermo Scientific 10566016) at 37 °C and 5% CO_2_. LNP with formulated siRNA were premixed with DMEM medium and added to the adherent cells in the 10 nM final concentration of siRNA. Cells were incubated for 2 and 4 days, with the repeat of the transfection protocol on the second day, with the following estimation of the efficacy of Mettl3 protein inhibition by Western blot.

### 2.3. RNA Isolation and RT-qPCR

Total RNA was isolated using TRIzol (ThermoFisher Scientific, Waltham, MA, USA), according to the manufacturer’s instructions. Then, ~0.5–1 μg of RNA was further treated with DNase I (ThermoFisher Scientific), supplied with RiboLock RNase Inhibitor (40 U/μL) to the final concentration 0.4 U/μL. For RT-qPCR, treated total RNA was used to synthesize cDNA using Maxima First Strand cDNA Synthesis Kit, followed by qPCR using PowerUp™ SYBR™ Green Master Mix (ThermoFisher Scientific). PCRs were performed using the primers listed in Supplementary Table S2. Gapdh mRNA was used as a control for the total RNA and cytoplasmic fraction, with u2 as a control for nuclear fraction.

### 2.4. Anti-m6A Immunoprecipitation

Immunoprecipitation (IP) of m6A-modified RNA using an EpiMark^®^ N6-Methyladenosine Enrichment Kit (E1610S, New England Biolabs, Ipswich, MA, USA) was carried out following a manufacturer’s protocol. Briefly, total RNA (~250 μg) was incubated with m6A antibody, bounded to Protein G Magnetic Beads (New England Biolabs, #S1430), in 1× reaction buffer (150 mM NaCl, 10 mM Tris-HCl, pH 7.5, 0.1% NP-40 in nuclease free water), according to manufacturer’s protocol. After extensive washing with 1× reaction buffer, m6A-modified RNA was eluted using Buffer RLT (Qiagen, Venlo, The Netherlands, #79216), purified and concentrated with Dynabeads MyOne Silane (Life Technologies, Carlsbad, CA, USA, #37002D), according to manufacturer’s protocol. Then, 47S pre-RNA, mRNA nucleolin, and A’-A0 RNA spacer fragments were analyzed in the eluted RNA by RT-qPCR using primers listed in Supplementary Table S2.

### 2.5. Formaldehyde-Crosslinked RNA-Immunoprecipitation (RIP)

One 10-cm plate with 90% confluent HEK293 cells was used to prepare each sample. Harvested cells, resuspended in 2 mL of phosphate buffered saline (PBS), were crosslinked by adding 143 μL of 37% methanol-free formaldehyde and incubated for 10 min at room temperature. Crosslinking was terminated by the addition of 685 μL of 2 M glycine solution in water. Cells were washed twice with ice-cold PBS, followed by centrifugation at 1000× *g* for 5 min at 4 °C. Cell pellets were resuspended in 1 mL of IP lysis buffer (50 mM HEPES (pH 7.5), 0.4 M NaCl, 1 mM EDTA, 1 mM DTT, 0.5% TritonX-100, 10% glycerol) with added 20 μL of 0.1 M phenylmethylsulfonyl fluoride (PMSF), 20 μL of complete protease inhibitor (50×), and 5 μL of RNase inhibitor (40 U/μL). The lysates were sonicated (10 s ON, 10 s OFF, amplitude 20 μm, 10 cycles; QSonica sonicator, amplitude 20%) and centrifugated at 14,000× *g* for 3 min. Supernatant with crosslinked protein-RNA complexes was subjected to IP overnight at 4 °C with an anti-Mettl3 (ab195352, Abcam, Cambridge, UK) or human IgG (control) antibody, bound to preblocked Sepharose G-Beads (ab193259, Abcam). Then beads were washed with the IP buffer (five times), followed by a wash with the RIP buffer (50 mM HEPES (pH 7.5), 0.1 M NaCl, 5 mM EDTA, 10 mM DTT, 0.5% Triton X-100, 10% glycerol, 1% SDS). Samples were incubated at 70 °C for 30 min, centrifugated at 1000× *g* for 5 min. RNA samples were extracted using TRIzol following the manufacturer’s protocol and analyzed by reverse transcription qPCR amplification using the primers listed in Supplementary Table S2.

### 2.6. Nascent RNA Purification and cDNA Preparation

Nascent RNA was prepared and purified from total RNA samples using Click-iT Nascent RNA Capture Kit (#C10365, Thermo Fisher) according to the manufacturer’s instructions. In brief, 0.5 mM ethylene uridine (EU) nucleotide was added to cell culture and incubated for 1 h. Then 5-EU-labeled RNA was purified and conjugated to biotin azide using Cu(I)-catalyzed azide-alkyne cycloaddition (CuAAC) click chemistry. Then biotinylated nascent RNA was purified using MyOne Streptavidin T1 magnetic Dynabeads (Life Technologies). Reverse transcription was done using cDNA synthesis reaction mix (random hexamer (6 μg) (Integrated DNA Technologies, Coralville, IA, USA), anchored oligo-(dT)20 (2 μg) (Integrated DNA Technologies), 10 mM dNTP mix (2 μL) (Life Technologies), and the buffer J (14 μL) from Click-iT Nascent RNA Capture Kit (Thermo Fisher) in a total volume 20 μL. Mix was added to the beads and incubated for 10 min at 70 °C, and then chilled on ice for 5 min. Next, a mix of 10× RT buffer (4 μL), 20 mM MgCl_2_ (8 μL), 0.1 M DTT (4 μL), 20 U/μL SuperaseIn (2 μL), and 200 U/μL SuperScript III Reverse Transcriptase (2 μL) (all from Life Technologies) was added to the mixture and incubated for 10 min at 25 °C, 50 min at 50 °C, and finally 5 min at 85 °C for first-strand cDNA synthesis. To digest RNA and release the first-strand cDNA, 2 U/μL Escherichia coli RNase H (2 μL) (Life Technologies) was added, followed by a 20-min incubation at 37 °C. The beads then were removed using a magnet and first-strand cDNA was used for second-strand cDNA synthesis by adding nuclease-free water (70 μL) (Life Technologies), second-strand buffer (30 μL) (Life Technologies), 10 mM dNTP mix (3 μL) (Life Technologies), 10 U/μL E. coli DNA Polymerase (4 μL) (New England Biolabs), and 10 U/μL E. coli DNA ligase (1 μL) (New England Biolabs). The mixture was then incubated for 2 h at 16 °C. Finally, double-stranded cDNA was purified using Agencourt AMPure XP beads (Beckman Coulter, Brea, CA, USA) and analyzed by RT-qPCR. 

### 2.7. Separation of Nuclear and Cytoplasmic Cell Fractions

For separation of cytoplasmic and nuclear fractions, cells were resuspended in a low-salt buffer (20 mM HEPES (pH 7.9), 10% glycerol, 1.5 mM MgCl_2_, 0.05% NP-40, and 0.4 U/μL RiboLock RNase Inhibitor (40 U/μL)). After intensive mixing, cells were kept on ice for 5 min and centrifuged at 4000 rpm for 5 min at 4 °C. Cytoplasmic fraction was collected as a supernatant. Remaining pellet was then resuspended in high-salt buffer (20 mM HEPES (pH 7.9), 10% glycerol, 1.5 mM MgCl_2_, 0.05% NP-40, 0.4 U/μL RiboLock RNase Inhibitor (40 U/μL), and 0.5 M NaCl), put on ice for 10–15 min and centrifuged at 12,000× *g* for 5 min at 4 °C to isolate supernatant with nuclear fraction. Then, total RNA was isolated using TRIzol either from cytoplasmic or nuclear fractions.

### 2.8. Fluorescent in Situ Hybridization (FISH)

Mettl3 knock-down (KD) and control cells were grown on microscopy glasses coated with poly-lysine for 4 days. Cells were washed twice with PBS and fixed with 4% PFA (Sigma Aldrich) at RT for 12 min. Thereafter, cells were washed with PBS twice for 10 min and permeabilized for 10 min in 70% ethanol. Then cells were washed by PBS for 10 min followed by two washes in “FA wash” buffer (40% formamide, 2.4× SSC (saline sodium citrate) buffer). Glasses with cells were placed in hybridization chamber and stained with custom RNA probes (Supplementary Table S3) in a hybridization buffer (0.4% BSA (Sigma Aldrich) in water, 44% volume of formamide, and 11% volume of SSC buffer) supplied with tRNA + ssRNA mix (5.5 μg/μL) overnight at 37 °C. After that, cells were washed twice with FA wash buffer, preheated up to 37 °C for 15 min, and once with PBS at RT for 10 min. Thereafter, the cells were stained with 4′,6-diamidino-2-phenylindole (DAPI) (Thermo Scientific) (300 nM) for 2 min, washed with PBS, and studied by confocal microscopy. Confocal microscopy was performed using Nikon A1+MP confocal imaging system using a Plan Apo 20×/0.75 Dic N objective (numerical aperture = 0.75, Nikon, Tokyo, Japan), Apo LWD 40×/1.15 S water immersion objective (numerical aperture = 0.15, Nikon) and Apo tirf 60×/1.49 DIC Oil immersion objective (numerical aperture = 0.49, Nikon). Images were scanned sequentially using 561-nm diode lasers in combination with a DM561-nm dichroic beam splitter. 

### 2.9. Estimation of rRNA Precursors’ Lifetime

A half-life of the rRNA precursors was measured in Mettl3 KD and control cells using an actinomycin D assay. Then, ~80,000 cells in 48-well plate were incubated with actinomycin D (20 μg/mL) for 1, 2, 5, 10, 15, or 20 min. Then cells were harvested with TRIzol reagent followed by RNA purification and RT–qPCR to monitor the decay of the RNA precursors. To estimate the processing rate of the A0 and A’ cleavage in control and Mettl3 KD cells, data were fitted to exponent using the function y(x) = exp[−K × (x − x0)], where −K is an exponential decay and x0 is the time offset (GraphPad Prism 6.0 software).

### 2.10. Western Blotting

Cell extracts were prepared using ~8 × 10^6^ Mettl3 KD and control cells after 4 days of Mettl3 inhibition resuspended in a radioimmunoprecipitation assay buffer (RIPA) Lysis and Extraction Buffer (Thermo Fisher Scientific) supplied with 0.05% Triton X-100, 1 mM dithiothreitol (DTT), 0.2 mM phenylmethylsulfonylfluoride (PMSF), and 1x Halt™ Protease Inhibitor Cocktail. Concentration of the protein lysates was determined by Bradford protein assay (Thermo Fisher Scientific). Cell extracts (15–50 µg) were denatured by heating at 95 °C 10 min and separated on 10% SDS—polyacrylamide gels together with a PageRuler Pre-stained Protein Ladder (Thermo Fisher Scientific). Proteins were transferred to nitrocellulose membranes (Bio-Rad) using a Mini Trans-Blot^®^ Cell and Criterion™ Blotter (Bio-Rad Laboratories Inc., Hercules, CA, USA) at 80 V for 40 min at RT. The remaining protein binding sites of the nitrocellulose paper were blocked by immersion in TBS/Tween (10 mM Tris–HCl, pH 7.5, 150 mM NaCl, 0.05% Tween 20) with 5% Bovine Serum Albumin (Sigma-Aldrich Corp.) at 4 °C overnight. The blocked filter was incubated with primary antibody (anti-Fibrillarin (C13C3, Cell Signaling, 1:1000 dilution, anti-β-Actin (MA1-140, Thermo Fisher Scientific, 1:5000 dilution), anti-Mettl3 (ab195352, Abcam, 1:1000 dilution), anti-XRN2 (#13760, Cell Signaling, 1:1000 dilution), and anti-Nucleolin (#14574, Cell Signaling, 1:1000 dilution)) for 1 h at room temperature. After washing with TBS/Tween, the appropriate secondary antibody was added for an additional 1 h. Clarity™ Western ECL Blotting Substrates were used to develop the blots.

### 2.11. Ligation of DNA Probes Using RNA Template 

RNA from KD or control cells was added to a ligation reaction mixture A consisting of 20 nM probe L, 20 nM probe R, and the 1× ligation buffer in 5 µL. Probes are listed in Supplementary Table S4. Mixture A was heated at 85 °C for 3 min and then incubated at 35 °C for 10 min, then the ligation reaction mixture B (0.9 U T4 RNA ligase 2 in 1× ligation buffer in 5 µL) was added. The final volume of the ligation reaction mixture was 10 μL. PCR master Mix (Thermo Scientific) was used for amplifying cDNA fragment using FORWARD and REVERSE primers: 5’-ATCACCGACTGCCCATAGAG-3’ and 5’-CCATCTCATCCCTGCGTGTC-3’. The PCR protocol used was 98 °C for 30 s, 20 cycles of 98 °C for 10 s, 64 °C for 30 s, and 72 °C for 30 s, and the final extension at 72 °C for 2 min. PCR mixtures were separated by electrophoresis in 3% agarose gel and analyzed in ChemiDoc imaging system (Bio-Rad).

### 2.12. Polysome Profiling

Mettl3 KD and control cells were grown in 10-cm Petri dish for 4 days. Cells were first washed with cycloheximide in PBS (100 ug/mL final) and lysed using polysome cell extraction buffer (20 mM Tris-HCl (pH 7.5), 250 mM NaCl, 1.5 mM MgCl_2_, 1 mM DTT, 0,5% Triton X-100, 100 ug/mL cycloheximide, 20 U/mL TURBO DNAse). Following cell lysis (10 min on ice), nuclei and cell debris were cleared by centrifugation at 13,000× *g* for 10 min at 4 °C. Equal amounts of lysates according to the absorbance at 260 nm were accurately put on the top of centrifuge tubes filled with 10–50% sucrose gradient (11 mL). Lysates were separated by centrifugation at 35,000× *g* for 3 h at 4 °C with max break (Beckman Coulter SW 41 Ti rotor) and final solutions were manually divided into 300-uL fractions using a single channel pipette (100–1000 uL). Absorbance of each fraction was measured at 254 nm in Costar UV 96-well plate using Varioscan spectrophotometer and normalized to the total absorption.

### 2.13. Bioinformatic Analysis

**Figure 1:**
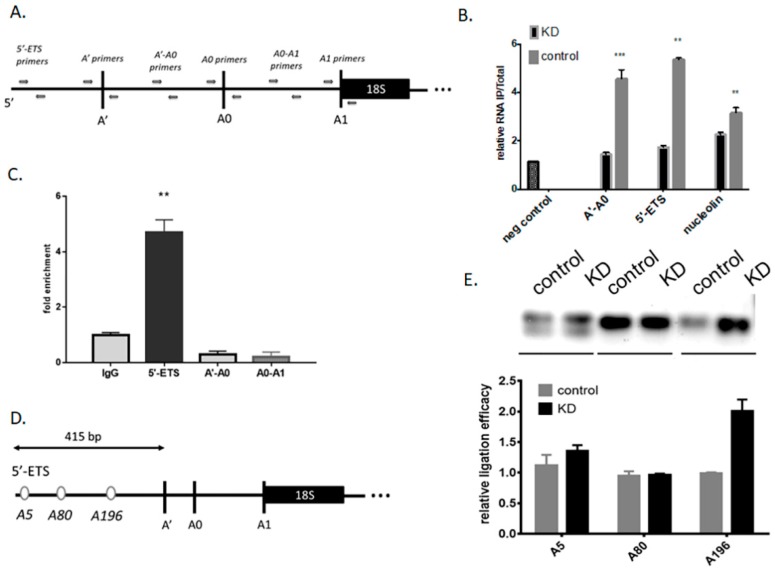
Characterization of RNA methyltransferase Mettl3 interactions with 5′-ETS of 47S pre-rRNA. (**A**) Scheme of the RT-qPCR primer locations on the pre-47S rRNA. (**B**) Relative amounts of rRNA fragments in immunoprecipitates obtained with anti-m6A antibodies from control and Mettl3 KD cells, quantified by RT-qPCR analysis (* *p* < 0.05, ** *p* < 0.01, *** *p* < 0.001). Negative control (unmethylated RNA from the EpiMark^®^ N6-Methyladenosine Enrichment Kit) A’-A0 (primers for amplification of A’-A0 spacer), 5′-ETS (primers for amplification of the part of 47S pre-rRNA before A’), nucleolin (m6A methylated mRNA based on MeT-DB2.0 browser data). (**C**) Enrichment of rRNA fragments in immunoprecipitates obtained with anti-Mettl3 antibodies from control cells, quantified by RT-qPCR analysis. (**D**) Scheme of 5′-ETS in rRNA with designated adenosines in the context of predicted DRACH sequences. (**E**) PCR analysis of T4 RNA ligase assay for pre-rRNA from control and Mettl3 KD cells (visualization of fragments separated using gel-electrophoresis and their relative quantification).

MeT-DB V2.0 Genome Browser (https://180.208.58.19/metdb_v2/html/genome_browser.php) and MeT-DB V2.0 TREW Database (https://180.208.58.19/metdb_v2/html/trew.php) were used for analysis of potential m6A sites in pre-rRNA. We identified alterations in methylation patterns within specific transcripts for Mettl3-KO and control tracks (p007_HeLa_KO_M3_ip and p007_HeLa_ctrl_ip, p009_HUMAN_siMETTL3_ip and p009_HUMAN_siControl_ip) using MeT-DB V2.0 [34]. We identified sites of Mettl3 methyltransferase interaction with specific transcripts using MeT-DB V2.0 TREW Database (https://180.208.58.19/metdb_v2/html/trew.php). Human 47S rRNA transcript sequence (NR_146144.1) was used to detect DRACH sequences (Figure 1C) within it in order to identify potential sites of interaction of 47S rRNA with Mettl3 enzyme. 

### 2.14. Statistical Analysis of the Experimental Data

All the diagrams are based on at least three independent experiments. Statistical data processing was performed using the GraphPad Prism software (version 8.3) with a two-sample t-test, as well as a two-way ANOVA analysis of variance or repeated-measures ANOVA and Sidak *t*-test. The data were considered statistically significant at *p* < 0.05.

## 3. Results

### 3.1. Mettl3 Methyltransferase Binds to 47S Pre-rRNA and Modifies Adenosine 196 

As Mettl3 methyltransferase is essential for eukaryotic cells (knockout is lethal [35]), we chose a RNAi-mediated knockdown approach to study if Mettl3 can contribute in pre-rRNA processing. We designed siRNA and found optimal conditions for Mettl3 knockdown (KD) in HEK293 cells, using siRNA targeting mRNA of firefly luciferase as a control. A second siRNA was used to assess false phenotypes due to off-target effects. Mettl3 protein was not detected by western blot at day 4 for at least two siRNA targeting Mettl3 mRNA (Supplementary Figure S1A,B). Therefore, these conditions were used in the next experiments. 

First, we verified the presence of m6A modification in the precursors of rRNA by m6A-immunoprecipitation followed by RT-qPCR analysis (scheme of the RT-qPCR primer locations on the pre-47S rRNA- Figure 1A). Comparison of m6A enrichment in control vs. Mettl3 KD cells allowed excluding of false positive immunoprecipitation of the very abundant pre-rRNA. Nucleolin mRNA is a known target for Mett3 and was used as a positive control in our study. We found that the amount of 5′-ETS and A’-A0 spacer of 47S pre-rRNA isolated by IP decreased in Mettl3 KD cells (Figure 1B). This result predicts the presence of m6A modified nucleotides in these parts of 47S pre-rRNA. These encouraging results pushed us to perform additional experiments to confirm the first conclusions and study the role of Mettl3 in pre-RNA processing. We performed RNA-immunoprecipitation from HEK293 cell extract with anti-Mettl3 antibodies followed by RT-qPCR analysis. Despite the m6A IP results (Figure 1B), we observed enrichment of only the 5′-ETS of 47S precursor rRNA in comparison to A’-A0 and A0-A1 spacers (Figure 1C). Thus, we concluded that Mettl3 interacted only with the 5′-ETS of 47S pre-rRNA and can methylate it.

At the next step we analyzed the sequence of 5′-ETS of 47S pre-RNA and predicted sites for adenosine methylation. Methyltransferase Mettl3 recognizes specific motifs called DRACH sequences (D = A/G/U, R = A/G, H = A/C/U) [36]. We found three possible positions for N6-methyladenosine in the 5′-ETS of 47S pre-rRNA (Figure 1D). Each possible methylation site was analyzed by a T4 RNA ligase assay [37]. In this assay, the presence of m6A opposite to the gap between 3′-OH and 5′-phosphate oligonucleotides strongly decreased the efficacy of ligation, and this can be further studied by PCR. Optimal experimental conditions were found using known probes for the m6A at position 2577 and A at position 2488 (Supplementary Figure S2A). We observed that downregulation of Mettl3 influenced on the efficacy of ligation only in the case of the adenosine at position 196 in the 5′-ETS of 47S pre-rRNA (Figure 1E). Thus, we demonstrated that methyltransferase Mettl3 interacted with the 47S precursor of rRNA and methylates adenosine 196 in the 5′-ETS. 

### 3.2. Knockdown of Mettl3 Methyltransferase Increases Rates of Pre-rRNA Maturation

**Figure 2:**
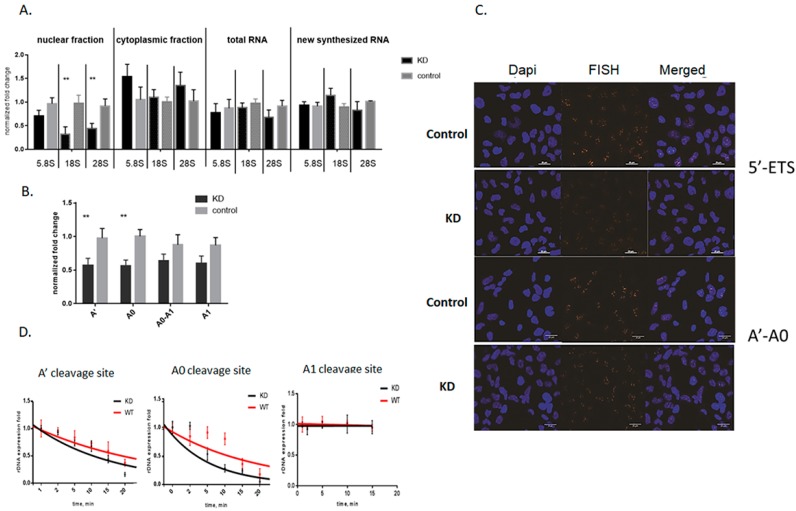
Changes in pre-rRNA processing after 4 days of Mettl3 KD. (**A**) RT-qPCR analysis of the mature 18S, 28S, and 5.8S rRNA in the nuclear and cytoplasmic fraction, total RNA, and nascent RNA in control and Mettl3 KD cells. Gapdh mRNA was used as a control for cytoplasmic fraction and total RNA, u2 (for the nuclear fraction) (* *p* < 0.05, ** *p* < 0.01). (**B**) Analysis of pre-rRNA cleavage efficacy by RT-qPCR at A’, A0 cleavage sites (primers were designed around cleavage site), and quantification of the A0-A1 RNA spacer in the total RNA (* *p* < 0.05, ** *p* < 0.01). (**C**) FISH analysis of 5′-ETS of 47S pre-rRNA, A’-A0 RNA spacer in control, and Mettl3 KD cells. DNA was stained with DAPI, and rRNA with Cy3-labeled probes (Table S3). (**D**) Estimation of the pre-RNA processing rate at A0 and A’ cleavage sites in control and Mettl3 KD cells by actinomycin D assay.

The m6A modification in pre-rRNA can have an effect on multiple cellular processes, such as rRNA maturation, ribosome assembly, and indirectly on the translation process [38,39]. First, we investigated whether Mettl3-mediated pre-rRNA methylation influences on the levels of mature rRNA. We quantified total and nascent 18S, 28S, and 5.8S rRNA from control and Mettl3 KD cells by RT-qPCR. Our results showed that Mettl3 downregulation does not noticeably influence on the levels of total and nascent mature rRNA (Figure 2A). However, m6A modifications can influence not only on the amount of RNA but also on the intracellular localization. So, we separated nuclear and cytoplasmic fractions of control and Mettl3 KD cells and estimated the amounts of all mature rRNA forms by RT-qPCR. We carefully controlled the quality of the fraction separation by measurement of snoRNA quantities using RT-qPCR (Supplementary Figure S2B). We found that in the case of Mettl3 KD cells, the level of the precursors contained 18S and 28S rRNA decreased in the nuclear fraction and slightly increased in the cytoplasmic fraction (Figure 2A). This fact can be a result either of increased rates of rRNA maturation or rRNA degradation or changes in the transport to cytoplasm. To distinguish between these three processes, we first estimated the levels of rRNA precursors during the maturation process in the total RNA by RT-qPCR (Figure 1A, scheme of primers). We found an increase in the cleavage efficacy at the A’ and A0 sites in the 47S and 45S precursors that resulted in a decrease of the amount of the A0-A1 fragment of pre-rRNA (47S, 45S, and 30S precursors) (Figure 2B). These results were reproduced using two alternative Mettl3 siRNA to exclude the probable off-target RNA downregulation by RNAi (Supplementary Figure S2C,D). To confirm these results, we quantified the 5′-ETS of 47S pre-rRNA and the segment between the A’ and A0 cleavage sites (A’-A0) by fluorescence in situ hybridization (FISH) and found a decrease of the fluorescent signal in the cell nucleus of Mettl3 KD cells (Figure 2C). Also, we quantified mature rRNAs (18S, 28S, 5.8S) by FISH and could not find any changes in comparison to control cells (Supplementary Figure S3A). U2 RNA was used as a negative control because this RNA was not influenced by Mettl3 KD. 

Next, we investigated the dynamics of pre-rRNA processing in Mettl3 KD cells after actinomycin D (ActD) treatment. ActD inhibits pre-rRNA synthesis, so one can selectively monitor processing and decay of previously transcribed rRNA intermediates [40]. We measured the fold changes of the cleavage efficacy at positions A’, A0, and A1 by RT-qPCR using primers located on both sides of the cleavage site (Figure 1A) [41]. After exponential fitting of the experimental curves, we observed significant increase of the A’ and A0 cleavage efficacy, which resulted in the decrease of the half-life for 47S and 45S pre-rRNA (Figure 2D) while the cleavage rate at the A1 site was unchanged. In the control sample, the half-life of 47S pre-rRNA was 16.0 ± 1.1 min while in the knockdown samples it was 11.4 ± 0.7 min. For the 45S pre-rRNA, the half-life was 9.9 ± 0.6 min in the control and 4.8 ± 0.5 min in the KD samples. Thus, the rate of rRNA processing was increased 1.5–2 times in the case of A’ and A0 cleavages of pre-rRNA in Mettl3 KD cells. Analysis of other cleavage sites in pre-rRNA did not reveal any significant changes (data showed for A1 site). Hence, Mettl3 KD influenced only on the 5′-ETS processing of 47S pre-rRNA. Finally, we characterized the translation activity in cells with Mettl3 depletion using polysome profiles. We found a slight increase of the portion of monosomes in Mettl3 KD cells that corresponds to previously published data (Supplementary Figure S3B). These results suggest that the surplus rRNA in the cytoplasmic fraction of KD cells serve to increase the number of ribosomes.

### 3.3. Influence of Mettl3 Methyltransferase Knockdown on Pre-rRNA Processing Factors

**Figure 3:**
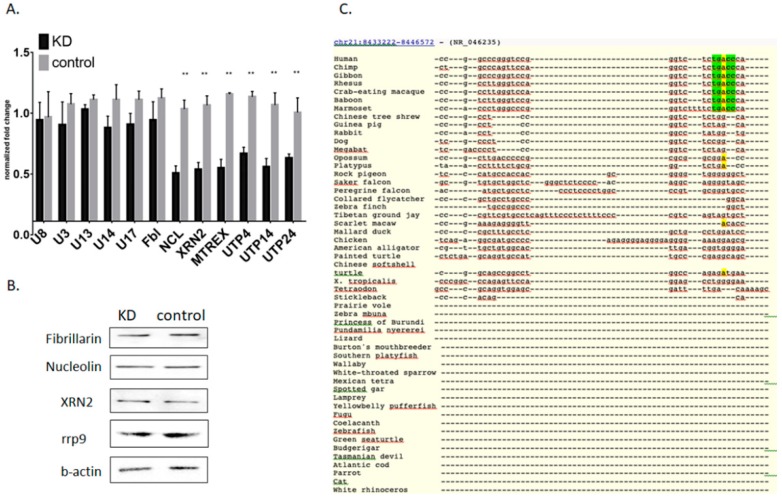
Changes of pre-RNA processing machinery after 4 days of Mettl3 KD. (**A**) Quantification of RT-qPCR data (* *p* <0.05, ** *p* < 0.01) and (**B**) Western blot analysis of the rRNA processing factors in control and Mettl3 KD cells. (**C**) Multiple sequence alignment of the 5′-ETS part with the modified m6A nucleotide (marked in yellow) in different species. Nucleotides of DRACH sequence are marked in green.

The changes observed in pre-RNA processing when Mettl3 is downregulated could be the result of deregulated snoRNA and protein factors participating in rRNA processing (Supplementary Figure S1C), as m6A methylation of mRNA can promote translation or be a signal for mRNA degradation [8]. Based on published data [42], analysis of mRNA using MeT-DB V2.0 browser [34], and Mettl3 interactome [43], we identified the presence of m6A sites in several mRNAs coding for pre-rRNA processing factors: Nucleolin, fibrillarin, chaperons UTPs, 5’-3’ Exoribonuclease 2 XRN2, exosome RNA helicase MTREX (Supplementary Figure S4). Thus, the decrease of Mettl3 methyltransferase may change the amounts of these proteins. We studied RNA and protein levels of the main participants of rRNA processing (snoRNAs U3, U8, U13, U14, and U17, fibrillarin, nucleolin, RNA helicase MTREX, RNAse Xrn2, UTPs factors in Mettl3 KD, and control cells) (Supplementary Figure S1C). The mRNA levels of MTEX, Xrn2, Utp4, Utp14, and Utp24 decreased twice, while snoRNAs U3, U8, U13, U14, U17, and nucleolin mRNA were not changed (Figure 3A). However, protein levels of fibrillarin, nucleolin, Xrn2, and rrp9 were unchanged in Mettl3 KD cells (Figure 3B). We performed our experiments at day 4 after initial RNAi downregulation of Mettl3, so we expected that significant decrease of Mettl3 influenced only on mRNA levels while protein levels of pre-rRNA processing factors were still unaffected. Thus, we propose that all observed effects were not caused by changes in the pre-rRNA processing machinery but by direct influence of m6A196. 

## 4. Discussion

Ribosome assembly in eukaryotes begins in the nucleus and begins with the maturation of pre-rRNA. The single large rRNA precursor synthesized by RNA polymerase I (47S in mammals) co-transcriptionally binds to a number of ribosomal proteins, ribosome maturation and assembly factors, and small nucleolar ribonucleoprotein complexes needed for pre-rRNA folding. During pre-rRNA maturation, external transcribed spacers (5′-ETS and 3′-ETS) and internal transcribed spacers (ITS) are sequentially eliminated through precisely controlled series of endo- and exonucleolytic cleavages [44], resulting in the formation of mature rRNA. Removal of transcribed spacers and nucleotide modification takes place concomitantly with the folding of pre-rRNAs and their assembly with ribosomal proteins. Modification of rRNA provides additional quality control mechanisms in pre-rRNA processing and ribosome biogenesis. Early occurring methylation of 2′-OH in ribose residues fine tunes folding of pre-rRNA and modulates binding to ribosomal proteins [45]. Also, RNA methyltransferases themselves or in complex with protein partners bind to rRNA during maturation and promote ribosome assembly [26]. All these processes contribute in the regulation and quality control of pre-rRNA cleavage. 

Initially, we hypothesized that Mettl3 activity can influence on rRNA processing as multiple RNA in the cell are targets of this methyltransferase. First, we found an unknown m6A modification site in the 5′-ETS of 47S pre-rRNA and confirmed that Mettl3 methyltransferase modifies adenine 196 in the 5′-ETS region. Knockdown of Mettl3 resulted in increased efficacy of the cleavage of the 47S precursors at positions A’ and A0 (Figure 2B), increased rates of rRNA processing (Figure 2D), and decreased amounts of 47S and 45S pre-rRNAs in the cell. We also found that downregulation of Mettl3 led to a decrease of mature rRNA amounts in the cell nucleus and a slight increase in the cytoplasm (Figure 2A). These results confirmed that Mettl3 KD influence on pre-RNA processing. However, m6A methylation of pre-rRNA can be just a secondary event, while alternative mechanisms other than direct input from m6A196 can accelerate processing. In order to test this, we first confirmed that the whole amount of 18S, 28S, and 5.8S rRNA in the cell is not changed in Mettl3 KD cells. Therefore, we can exclude enhanced pre-rRNA transcription as a stress response to the decrease of total m6A levels. Second, we studied changes in the pre-rRNA processing machinery, as many mRNA are m6A modified and some mRNA of processing factors can be found in the interactome profile of Mettl3 [10,43,46]. In Mettl3 KD cells, we revealed the decrease of mRNA level for the majority of the participants involved in the processing machinery of 5′-ETS region (UTP4, UTP14, UTP24, Nucleolin), ITS-2, and 3′-ETS regions adjacent to 28S rRNA (MTREX), but at the same time protein levels of these factors remained unchanged. U3, U8, U13, U14, and U17 snoRNAs were not changed either. We concluded that in our experimental conditions, the pre-rRNA processing machinery was intact despite knockdown of Mettl3. Thus, decreased adenosine methylation or Mettl3 binding or both events in the 5′-ETS region itself can be a possible driving force for increased pre-rRNA processing. We analyzed the outcome of increased rates of rRNA processing on ribosome biogenesis and found a slight increase of the 80S monosomes (the final destination for mature rRNA) in cells with Mettl3 KD. The polysome profiles obtained correlate with published data developed for the stable Mettl3 KD cell line [47]. Alternative results demonstrated a decrease in 80S ribosomes levels and increase in 60S ribosomal subunits after Mettl3 RNAi-mediated downregulation with a less efficient KD [48,49]. So, we propose that the effect of Mettl3 KD on the polysome profile depends on the time and efficacy of Mettl3 downregulation. Also, the increased portion of 80S ribosomes may serve as an additional storage space for the whole translation machinery in the cell to improve translation efficacy for mRNA with decreased levels of expression. In our case, efficient Mettl3 downregulation (no protein observed by WB on day 4) together with the short half-life of pre-rRNA and prolonged stability of pre-rRNA processing machinery allowed us to study the role of m6A 196 in 5′-ETS region in optimal experimental conditions. 

Additionally, we showed that identified DRACH sequence in 5′-ETS region is found and conserved only in primates (Figure 3C and Supplementary Figure S5). We propose that the evolution of 47S pre-rRNA resulted in an additional quality control for initial stages of rRNA processing. This fact can be driven by the increased complexity of ribosome biogenesis in primates, including regulatory networks that modulate ribosome assembly and function [50], and high risks coming from dysregulation of this process. Qualitative changes in the nucleotide modifications during the ribosome assembly can also correlate with the proliferation of the cell and dictate its transformative potential. However multiple functions of Mettl3 methyltransferase lead to the lack of correlation, as Mettl3 possesses pro-and anti-oncogenic roles in different types of cancer [51]. Probably, pre-rRNA modification by Mettl3 should be taken into consideration in the regulation of cellular processes and development of cancer.

**Figure 4:**
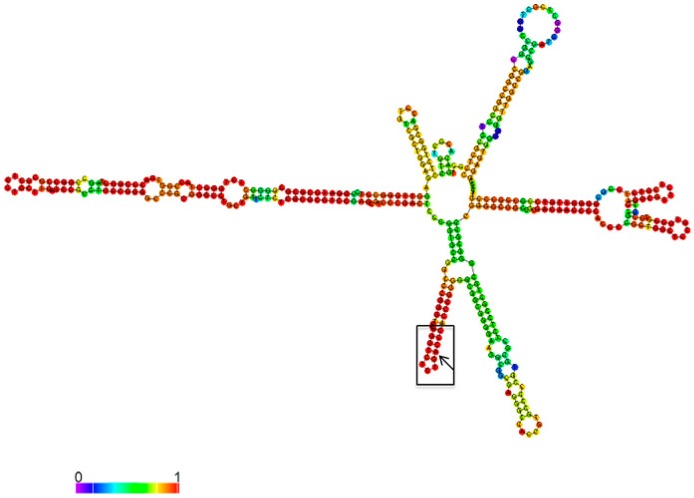
Predicted secondary structure of the first 415 bp of 5′-ETS in 47S pre-rRNA. Black square marks the helix with adenosine 196 (pointed by black arrow).

A196 in the 5′-ETS of 47S pre-rRNA is located in the stable duplex region (predicted by Vienna software, Figure 4), indicating that N6-methylation might disrupt or unwind local secondary structures. Based on our m6A-immunoprecipitation results, the ratio of methylated adenosines in 47S pre-rRNA was close to stoichiometric. As a result, a m6A-switch mechanism [19,20] can alter binding of processing factors or decrease accessibility of 5′-ETS for degradation by ribonucleases. We found using the RIP method that methyltransferase Mettl3 binds with the 5′-ETS of 47S pre-rRNA (Figure 1). Mettl3 methyltransferase predominantly localizes in the cell nucleus [52], where the rRNA maturation process takes place [44], so Mettl3 can contribute in pre-rRNA processing. Not all protein factors required for ribosome synthesis and directly associated with pre-ribosomal complexes are known yet, and the exact “step-by-step” human rRNA maturation process is still undiscovered [50,53]. Functions of many m6A modifications in RNA are still poorly understood yet indispensable for cell viability or organism development. Further studies are likely to provide more detailed information on the molecular mechanisms of m6A involvement in pre-rRNA maturation.

## 5. Conclusions

In conclusion, we demonstrated that RNA methyltransferase Mettl3 interacts with the 5′-ETS of 47S pre-rRNA and modifies adenosine 196. Downregulation of Mettl3 increases efficacy of pre-rRNA cleavage at positions A’ and A0, which leads to the decrease of 47S and 45S pre-rRNA amounts in the cell and increase of mature rRNA in the cytoplasm. As the processing machinery (U3, U8, U13, U14, and U17 snoRNA, and fibrillarin, nucleolin, Xrn2, and rrp9 proteins), rRNA degradation rates and total amount of mature rRNA in the cell are unchanged and A196 methylation leads to upregulation of pre-rRNA processing rates. Adenosine 196 is conserved in primates and may participate in a quality control mechanism for initial stages of pre-rRNA maturation and ribosome biogenesis. 

## Supplementary Materials

The following are available online at https://www.mdpi.com/2073-4409/9/4/1061/s1: Supplementary Table S1, List of Mettl3 siRNA used in the study. Supplementary Table S2, List of primers used in the study. Supplementary Table S3, List of FISH probes used in the study. Supplementary Table S4, List of probes for T4 RNA ligation assay used in the study. Supplementary Figure S1, (A) Determination of the Mettl3 siRNA efficacy by RT-qPCR (* *p* < 0.05, ** *p* < 0.01). 1–6 are the numbers of siRNA from Supplementary Table S1. (B) Determination the efficacy of the Mettl3 protein inhibition by Western blot. (C) Schematic representation of the human pre-rRNA processing with the cleavage sites. Supplementary Figure S2. (A) Gel electrophoresis of PCR analysis (18, 20, and 24 cycles of PCR) of T4 RNA ligase assay for the known m6A and A positions in Malat1 RNA. (B) Control of the quality of the cell fractions separation by RT-qPCR snoRNAs and mRNA Gapdh measurement. RT-qPCR analysis of the A’ (C) and A0 (D) cleavage efficacy in cells with Mettl3 KD using siRNA-3 and siRNA-4 (* *p* < 0.05). Supplementary Figure S3. (A) FISH analysis of mature rRNAs and U2 snRNA in control and Mettl3 KD cells. DNA is stained with DAPI, rRNA with Cy3-labeled probes. (B) Polysome profiling for control and Mettl3 KD cells. Supplementary Figure S4, Analysis of Mettl3-dependent m6A residues (highlighted in yellow) in UTP14 and nucleolin mRNA by MeT-DB V2.0 browser 31. Supplementary Figure S5, Multiple sequence alignment of 5′-ETS parts with highlighted m6A nucleotide (yellow) and DRACH sequence (green) in different species.
