# Supplementary material for: Modification of Adenosine196 by Mettl3 Methyltransferase in the 5’-External Transcribed Spacer of 47S Pre-rRNA Affects rRNA Maturation
Source: Cells. 2020 Apr 24;9(4):1061. doi: 10.3390/cells9041061 (PMC7226171; doi:10.3390/cells9041061)
Supplement: Supplementary file 1 [file cells-09-01061-s001.pdf]

cells

Supplementary Information

Modification of Adenosine196 by Mettl3 Methyltransferase in the 5'-External Transcribed Spacer of 47S Pre-rRNA Affects rRNA Maturation

Olga Sergeeva [1,*,†], Philipp Sergeev [1,2,†], Pavel Melnikov [3], Tatiana Prikazchikova [1], Olga Dontsova [1,4] and Timofei Zatsepin [1,4]

1 Skolkovo Institute of Science and Technology, Skolkovo, 121205 Moscow, Russia; Filipp.Sergeev@skoltech.ru (P.S.); T.Prikazchikova@skoltech.ru (T.P.); o.dontsova@skoltech.ru (O.D.); t.zatsepin@skoltech.ru (T.Z.)
2 Institute for Molecular Medicine Finland, Helsinki Institute of Life Science, University of Helsinki, FI-00014 Helsinki, Finland
3 Serbsky National Medical Research Center for Psychiatry and Narcology, Kropotkinsky Lane 23, 119034 Moscow, Russia; proximopm@gmail.com
4 Lomonosov Moscow State University, Department of Chemistry, 119992 Moscow, Russia
* Correspondence: o.sergeeva@skoltech.ru; Tel.: +79263880865
† Equal contribution.

Received: 3 April 2020; Accepted: 21 April 2020; Published: date

Supplementary Table S1. List of Mettl3 siRNA used in the study.

Name	Sequences, 5′-3′
1	uuAuuuGGGuGAAGAcAAATsT
	UUuGUCUUcACCcAAAuAATsT
2	AcuucAGAcGAAuuAucAATsT
	UUGAuAAUUCGUCUGAAGUTsT
3	uGuuGAAAAAuuucGcucuTsT
	AGAGCGAAAUUUUUcAAcATsT
4	ccAAGGAAcAAuccAuuGuTsT
	AcAAUGGAUUGUUCCUUGGTsT
5	GGAAcAAuccAuuGuuGAATsT
	UUcAAcAAUGGAUUGUUCCTsT
6	uGAAcGGGuAGAuGAAAuuTsT
	AAUUUcAUCuACCCGUUcATsT
control	cuuAcGcuGAGuAcuucGATsT
	UCGAAGuACUcAGCGuAAGTsT

Upper case – ribonucleotide, lower case – 2′-O-methylribonucleotide, s –phosphothioate group.

Supplementary Table S2. List of primers used in the study.

Name	Forward, 5′-3′	Reverse, 5′-3′
47S (5′-ETS)	TCGCTGGAGAGGTTGGGCCT	TCGGACGCGCGAGAGAACAGCA

A′	GTGTCAGGCGTTCTCGTCTCC	AGCGAGAAGGACGGTCCCGTT
A′-A0	GGTTTGCGCGAGCGTCGGCT	CAAACCGCCTCGAACCCCACAC
A0	GGCTCGTCGCCTACTGTG	CCAACCCGCTGGGAACG
A0-A1	GTGTTTTCCTGGTGGCCCGGC	GTTCGGTCCCAGGCGGGG
A1	GCCGCGCTCTACCTTACCTACCT	GCGAGCGACCAAAGGAACCATAACTG
18S	GGGGCCCGAAGCGTTTACTTTG	CAAGAATTTCACCTCTAGCGGCGC
5.8S	ACTCGGCTCGTGCGTC	GCGACGCTCAGACAGG
28S	AGAGGTAAACGGGTGGGGTC	GGGGTCGGGAGGAACGG
U8	ATCCTTACCTGTTCCTCCTCC	GGGTGTTGCAAGTCCTGA
UTP4	GTCCCTGTGACATCTAACAGCAGT	GCCTCCAGATATCAGCGCTGTT
UTP14	GCACCAAAACAGTGGGAAATGGG	TCTTCCTCACTCTCAGAGGCTACC
UTP24	GAATCTGTGCAGGGGGAAGCATATT	CTCTCCTCCTTTCCCAGGCATTGTA
MTREX	GTTTTGGTGCATCTCCTGTCTGC	GATGCCGTCAGGAAAACGTTTCTG
XRN2	CAGTGAACCTGAGCCAGAGGAT	GACTGCACAACTTTCCGACGGA
NCL	CCAGCCATCCAAAACTCTGT	TAACTATCCTTGCCCGAACG
GAPDH	TGCACCACCAACTGCTTAGC	GGCATGGACTGTGGTCATGAG
β-Actin	AGAGCTACGAGCTGCCTGAC	AGCACTGTGTTGGCGTACAG
FBL	CCACACAAATACCGCATGCTCATC	GTCAATGCAGTTGGCCTTAATGGA
METTL3	CGGAACCAGCAAAGGAGCCAGCCAAG	GTAGTATTTAATAGCTCTAGGATC

Upper case—2′-deoxynucleotide.

Supplementary Table S3. List of FISH probes used in the study.

FISH probe	Sequence, 5′-3′
47S	agacgagaacgccugacacgcacggcac-sCy3
A′-A0	cgcuagagaaggcuuuucuc-sCy3
18S	accagacuugcccuccaaug-sCy3

Lower case—2′-O-methylribonucleotide, sCy3—sulfo-Cyanine 3 dye.

Supplementary Table S4. List of probes for T4 RNA ligation assay used in the study.

Position	L probe, 5′-3′	R probe, 5′-3′
A5	p-CAGCTCTATGGGCAGTCGGTGAT	CCATCTCATCCCTGCGTGTC AGAGGACAGCGTgu
A80	p-CACCGGTAGGCCATCTATGGGCAGTCGGTGAT	CCATCTCATCCCTGCGTGTC CGGCTAGCCGGgu
A196	p-CAGAGACCCGGACTCTATGGGCAGTCGGTGAT	CCATCTCATCCCTGCGTGTC GCCGCCCCCGGgu
A2577	p-CCAATGCAAAAACTCTATGGGCAGTCGGTGAT	CCATCTCATCCCTGCGTGTCCTTAACTCAAAgu
A2488	p-ATATTTAAGGCTCTATGGGCAGTCGGTGAT	CCATCTCATCCCTGCGTGTCAACTAAGCTAcu

Upper case—2'-deoxynucleotide, lower case—ribonucleotide, p—phosphate group.

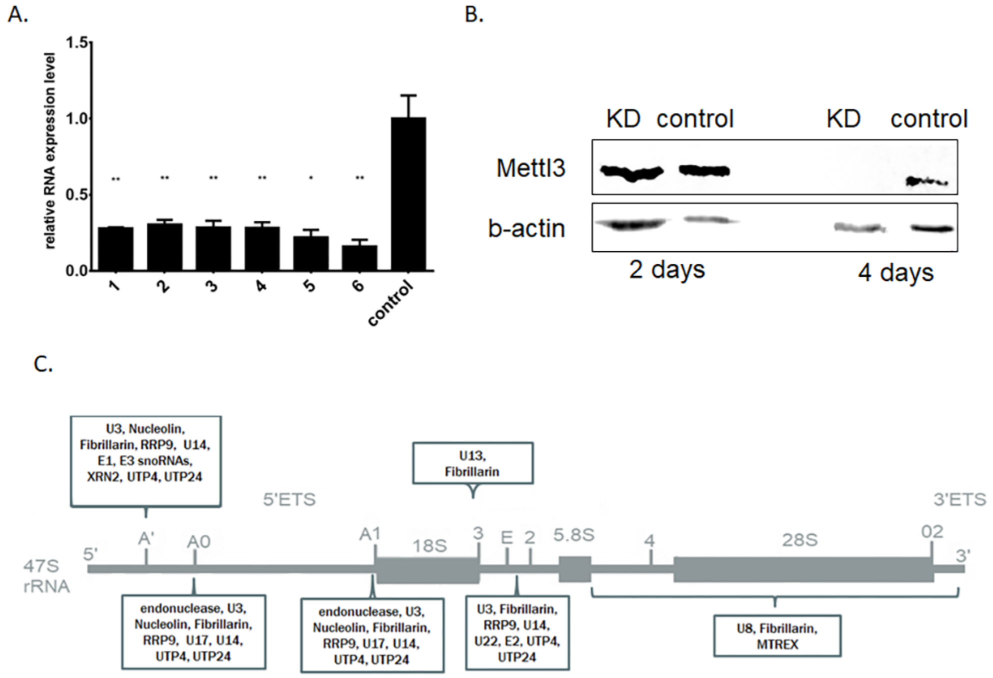

Figure S1. A. Determination of the Mettl3 siRNA efficacy by RT-qPCR (* $p < 0.05$, ** $p < 0.01$). 1–6 are the numbers of siRNA from Suppl. table 1. B. Determination the efficacy of the Mettl3 protein inhibition by Western-blot. C. Schematic representation of the human pre-rRNA processing with the cleavage sites.

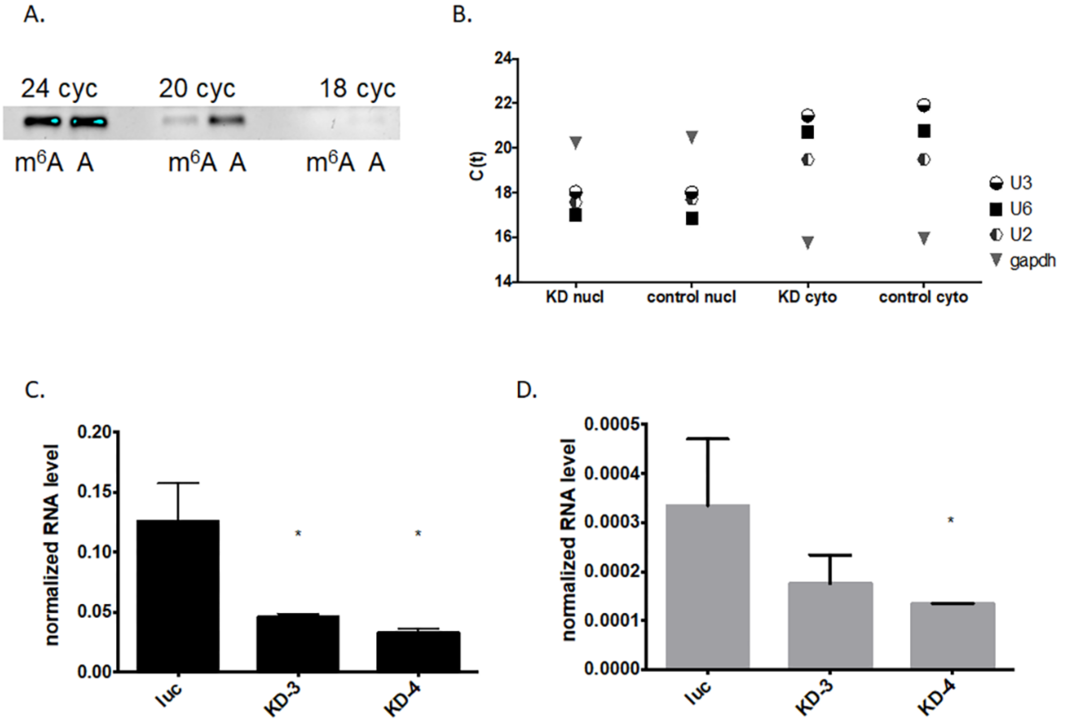

Figure S2. A. Gel electrophoresis of PCR analysis (18, 20 and 24 cycles of PCR) of T4 RNA ligase assay for the known m6A and A positions in Malat1 RNA. B. Control of the quality of the cell fractions separation by RT-qPCR snoRNAs and mRNA Gapdh measurement. RT-qPCR analysis of the A′ (C.) and A0 (D.) cleavage efficacy in cells with Mettl3 KD using siRNA-3 and siRNA-4 (* $p < 0.05$).

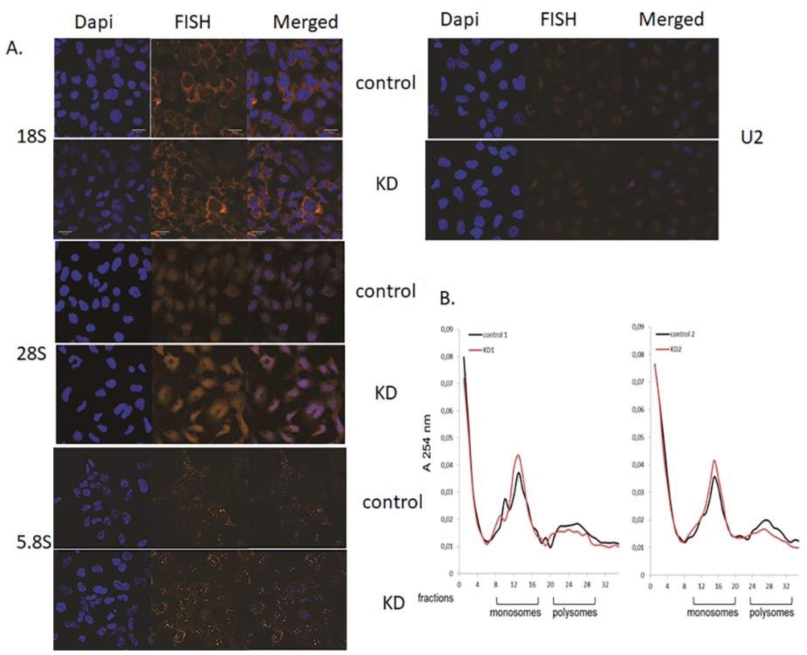

Figure S3. A. FISH analysis of mature rRNAs and U2 snRNA in control and Mettl3 KD cells. DNA is stained with DAPI, rRNA with Cy3-labeled probes. B. Polysome profiling for control and Mettl3 KD cells.

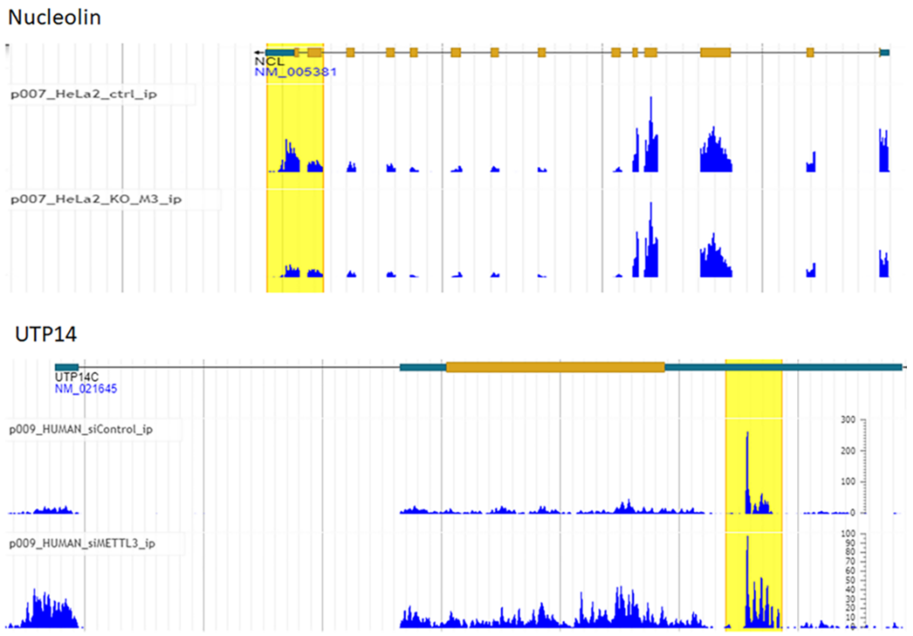

Figure S4. Analysis of Mettl3-dependent m6A residues (highlighted in yellow) in UTP14 and nucleolin mRNA by MeT-DB V2.0 browser 31. .

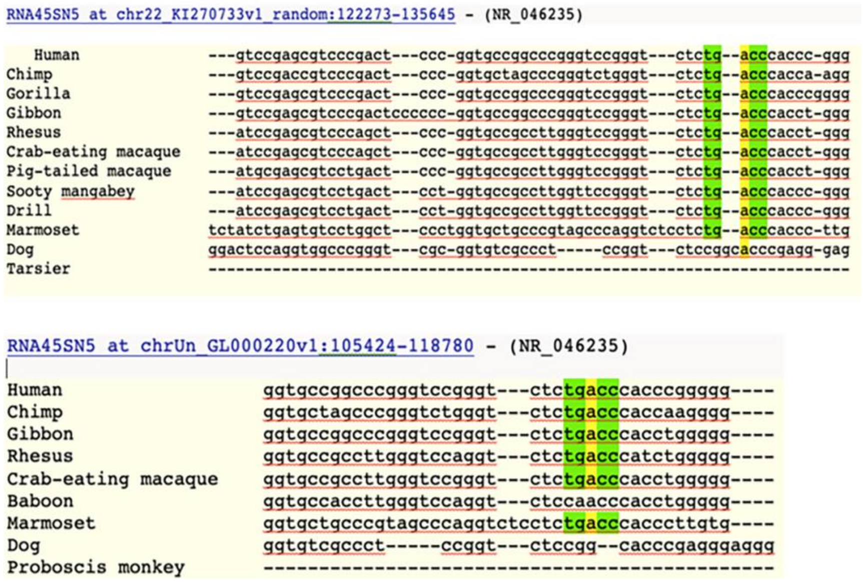

Figure S5. Multiple sequence alignment of 5′-ETS parts with highlighted m6A nucleotide (yellow) and DRACH sequence (green) in different species.

© 2020 by the authors. Licensee MDPI, Basel, Switzerland. This article is an open access article distributed under the terms and conditions of the Creative Commons Attribution (CC BY) license (http://creativecommons.org/licenses/by/4.0/).
